# An unusual presentation of penile fracture with complete transection of urethra: a case report

**DOI:** 10.1093/jscr/rjae290

**Published:** 2024-05-04

**Authors:** Vasanth Dunna, Srinivasa Rao Giduturi, K S N Chary, L V Simhachalam Kutikuppala, Anna Mary Jose, Varshitha Golla

**Affiliations:** Department of Urology, Citizens Speciality Hospital, Hyderabad, Telangana 500019, India; Department of Urology, NRI Medical College and General HospitalChinakakani, Andhra Pradesh 522503, India; Department of Urology, Citizens Speciality Hospital, Hyderabad, Telangana 500019, India; Department of General Surgery, Dr YSR University of Health Sciences, Vijayawada, Andhra Pradesh 520008, India; Department of Surgery, Datta Meghe Institute of Medical Sciences, Wardha, Maharashtra 442004, India; Department of Surgery, International School of Medicine (ISM), Bishkek 750065, Kyrgyzstan

**Keywords:** complete transection of urethra, penile fracture, urological emergency

## Abstract

Penile fracture is one such urologic emergency that occurs when the penis is struck bluntly during sexual activity, and in less than 5–10% of cases, the concurrent urethral damage is evident, but complete transection is very rare. A 37-year-old male presented with a history of ‘snap’ sound and immediate detumescence of penis during intercourse, when he fell and hit the pubic bone of his partner. There was acute retention of urine, an attempt to pass a catheter failed and the patient underwent supra-pubic catheterization. On examination, there was classical ‘eggplant deformity’ of the penis with blood at the tip of the meatus. MRI showed a tunical tear on both sides at the penoscrotal junction with indistinct urethra and extensive hematoma in the proximal penile shaft. Surgical management was successfully done by anastomotic urethroplasty and cavernosal repair.

## Introduction

Penile fracture is one such urologic emergency that is characterized by traumatic rupture of the tunica albuginea along with corpora cavernosa [1]. It occurs when the penis is struck bluntly during sexual activity and in less than 5–10% of cases, the concurrent urethral damage is evident, but complete transection is very rare [2]. An intra-corporeal pressure of 1500 mmHg or more is needed during erection to tear tunica albuginea [3]. Due to potential complications, such as the development of strictures and extravasation of urine, it is essential to investigate the degree of urethral damage in the penile fracture as soon as possible [3, 4]. We present a case report of penile fracture with complete urethral transection and its successful surgical outcome. We describe the presentation, diagnostic workup and management of a patient who presented with a fractured penis with complete transection of the urethra.

## Case presentation

**Figure 1:**
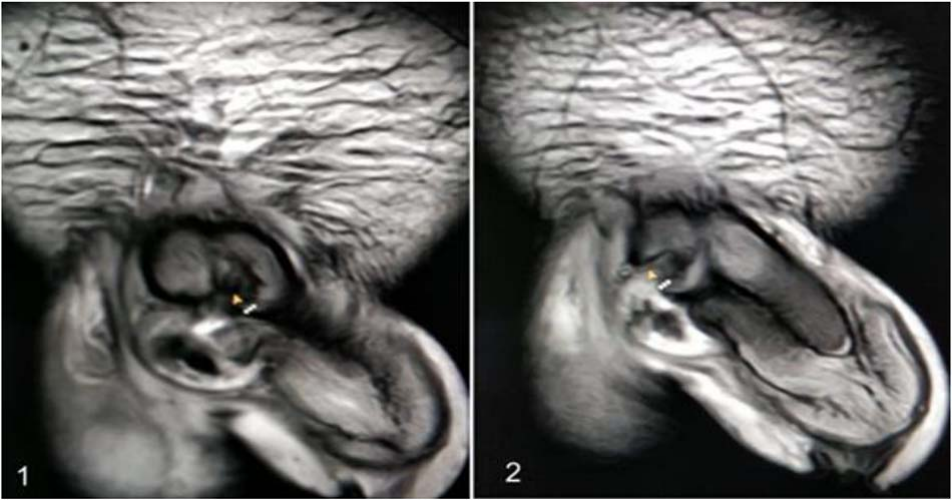
An MRI with (1) left corporeal tear on the left side with surrounding hematoma and (2) right corporeal tear with hematoma and disruption of urethra.

**Figure 2:**
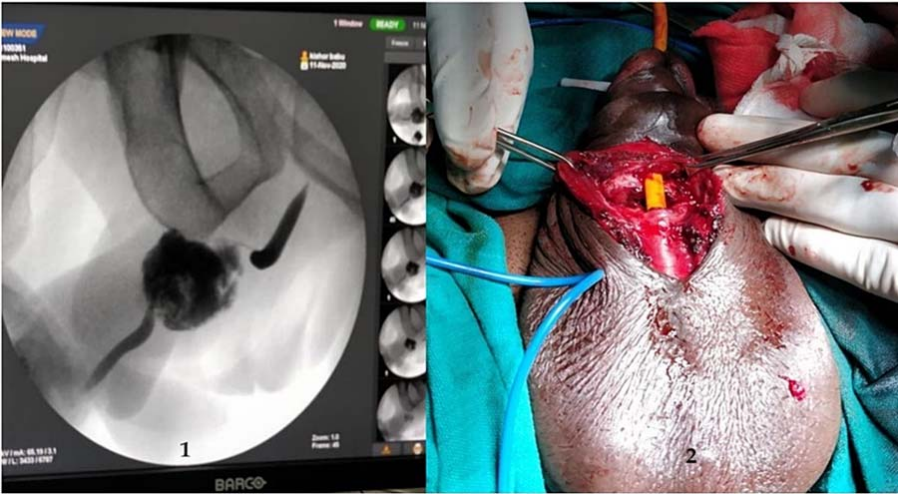
(1) On table RGU showing evidence of partial urethral disruption and (2) total transection of proximal urethra on exploration with penoscrotal vertical incision.

**Figure 3:**
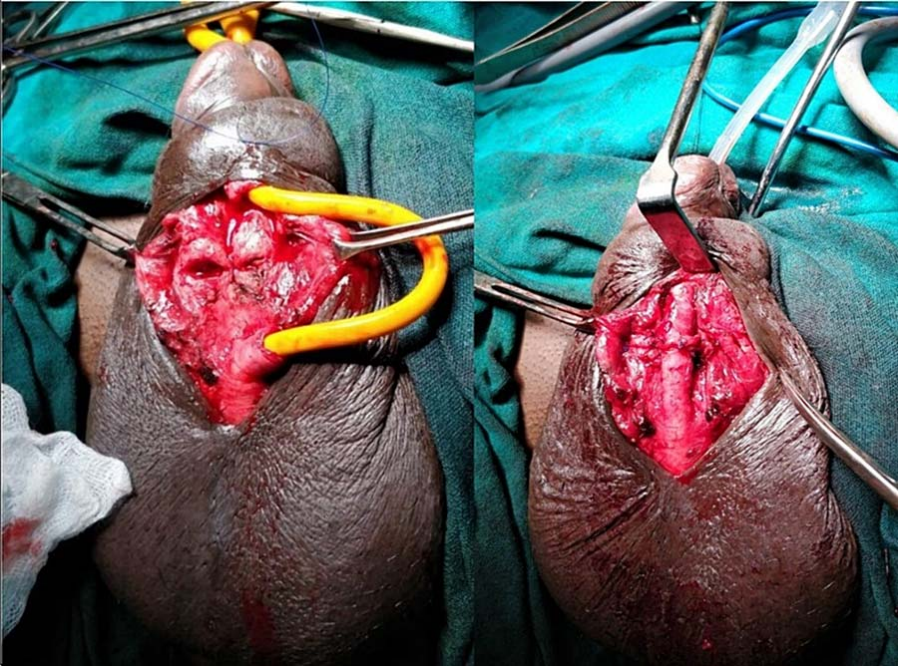
Reconstruction of midline septum with mobilized and anastomosed penile urethral ends.

**Figure 4:**
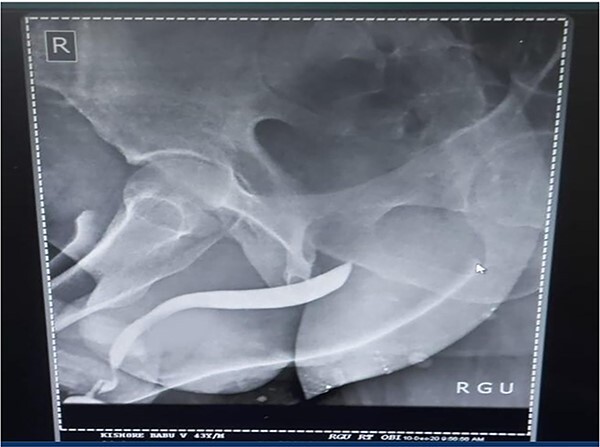
Follow-up RGU done after 3 months showing no evidence of stricture.

A 37-year-old male presented with a history of a ‘snap’ sound and immediate detumescence of penis during intercourse, when he fell and hit the pubic bone of his partner. There was generalized swelling and pain in the penis. There was acute retention of urine, an attempt to pass a catheter failed and the patient underwent supra-pubic catheterization. On examination, there was classical ‘eggplant deformity’ of the penis with blood at the tip of the meatus. MRI showed a tunical tear on both sides at the penoscrotal junction with indistinct urethra and extensive hematoma in the proximal penile shaft (Fig. 1). With the diagnosis of a fractured penis and possible urethral injury, after proper counselling, the patient was taken up for surgery. On the table, a retrograde urethrogram (RGU) was done showing evidence of partial urethral disruption. On exploration with penoscrotal vertical incision, there was total transection of proximal penile urethra, and a 1.5 cm tunical injury of corpus cavernosum on both sides ventrally (Fig. 2). There was extensive hematoma. Corpora sutured with 4.0 prolene after reconstructing the midline septum. Urethral ends mobilized and anastomosed with 3.0 vicryl in a single layer with minimal spatulation (Fig. 3). The patient was discharged on the 5th post-operative day; Foley catheter was removed on day 21. Follow-up RGU done after 3 months showed no evidence of stricture (Fig. 4).

## Discussion

Penile fracture is an uncommon urological emergency that occurs when the tunica albuginea in an erect penis rupture due to excessive external bending stress during sexual activity and the injury is most caused at the time of coitus [1, 2]. Other less common causes are masturbation and the practice of Taqaandaan [3, 4]. In most cases, a penile fracture results in a cracking sound, fast detumescence, abrupt enlargement and ecchymosis of the penis, which gives it the appearance known as ‘eggplant deformity’ [5]. The rupture occurs more often in the proximal shaft and is located ventrally in coital injuries. In our case, there was a bilateral corporal tear. Literature states that bilateral corporal ruptures are often accompanied by urethral injury [6]. Urethral rupture, although infrequent, has an incidence that varies between 1 and 38%. This rupture may be partial or complete [7, 8]. Blood in the meatus, hematuria and voiding symptoms are highly indicative of urethral rupture, but the absence of these findings does not exclude urethral lesions [9]. Our patient presented with swelling, pain and acute retention of urine, which was followed by a failed attempt at urethral catheterization and on examination of the penis, we found blood at the tip of the meatus. All these findings yielded a high suspicion of urethral injury and we decided to perform an RGU. In suspected cases of urethral injury, RGU may demonstrate contrast leakage at the lesion site and reveal the exact point of urethral injury. However, RGU can show false negative results in up to 28.5% of cases [10]. In this case report, RGU could only show evidence of a partial rupture but, intraoperatively, there was found to be a complete transection of the proximal penile urethra. Compared to the traditional non-operative options, surgical restoration of the tear has proven to provide patients with the greatest results in terms of both sexual function, cosmetic appearance and fewer complications [9, 11, 12]. Penile degloving using a circumferential or subcoronal incision is the most used technique followed by penoscrotal and longitudinal incisions directly over the hematoma. The sub-coronal degloving incision is useful in cases with significant penile swelling or extensive hematomas, as well as in cases where the location of tunical rupture cannot be determined clinically or radiographically, or when the rupture is located dorsally [13]. Major disadvantages of the sub coronal degloving incision are the extensive dissection of Buck’s fascia and its risk of distal penile/preputial skin necrosis. In this case, we performed a penoscrotal incision. We chose this technique because it gives easy exposure to the base of the corpora cavernosa where fractures tend to occur more often and in addition would give us direct access to the urethral transection. With the penoscrotal incision, we have the flexibility of extending the incision and can also avoid futile dissection in the buck’s fascia [13]. We reconstructed the midline septum and closed the corpora using 4.0 prolene. Urethral ends were mobilized and anastomosed with 3.0 vicryl in a single layer with minimal spatulation. The patient was discharged on the 5th postoperative day, and the Foley catheter was removed on day 21. A follow-up RGU performed after 3 months showed no evidence of stricture.

## Conclusion

Complete transection of the urethra in a case of penile fracture is an infrequent presentation. Evaluation by history, presenting symptoms, imaging and retrograde urethrography can establish the diagnosis. Surgical management can be successfully done by anastomotic urethroplasty and cavernosal repair, with better outcomes and minimal complications.

## References

[1] Sharma AP , Narain TA, Devana SK, et al. Clinical spectrum, diagnosis, and sexual dysfunction after repair of fracture penis: is no news good news? Indian J Urol 2020;36:117–22. 32549663 10.4103/iju.IJU_333_19 PMC7279096

[2] Bali RS , Rashid A, Mushtaque M, et al. Penile fracture: experience from a third world country. Adv Urol 2013;2013:708362. 23956740 10.1155/2013/708362 PMC3730138

[3] El Atat R , Sfaxi M, Benslama MR, et al. Fracture of the penis: management and long-term results of surgical treatment. Experience in 300 cases. J Trauma 2008;64:121–5. 18188109 10.1097/TA.0b013e31803428b3

[4] Zargooshi J . Sexual function and tunica albuginea wound healing following penile fracture: an 18-year follow-up study of 352 patients from Kermanshah, Iran. J Sex Med 2009;6:1141–50. 19138357 10.1111/j.1743-6109.2008.01117.x

[5] Koifman L , Cavalcanti AG, Manes CH, et al. Penile fracture -experience in 56 cases. Int Braz J Urol 2003;29:35–9. 15745465 10.1590/s1677-55382003000100007

[6] Hoag NA , Hennessey K, So A. Penile fracture with bilateral corporeal rupture and complete urethral disruption: case report and literature review. Can Urol Assoc J 2011;5:E23–6. 21470546 10.5489/cuaj.10055 PMC3104433

[7] Derouiche A , Belhaj K, Hentati H, et al. Management of penile fractures complicated by urethral rupture. Int J Impot Res 2008;20:111–4. 17673928 10.1038/sj.ijir.3901599

[8] Muentener M , Suter S, Hauri D, Sulser T. Long-term experience with surgical and conservative treatment of penile fracture. J Urol 2004;172:576–9. 15247735 10.1097/01.ju.0000131594.99785.1c

[9] Amer T , Wilson R, Chlosta P, et al. Penile fracture: a meta-analysis. Urol Int 2016;96:315–29. 26953932 10.1159/000444884

[10] Mydlo JH , Hayyeri M, Macchia RJ. Urethrography and cavernosography imaging in a small series of penile fractures: a comparison with surgical findings. Urology 1998;51:616–9. 9586616 10.1016/s0090-4295(97)00701-2

[11] Ibrahiem el-HI, el-Tholoth HS, Mohsen T, et al. Penile fracture: long-term outcome of immediate surgical intervention. Urology 2010;75:108–11. 19896174 10.1016/j.urology.2009.08.057

[12] Zargooshi J . Penile fracture in Kermanshah, Iran: report of 172 cases. J Urol 2000;164:364–6. 10893586

[13] Minor TX , Brant WO, Rahman NU, Lue TF. Approach to management of penile fracture in men with underlying Peyronie’s disease. Urology 2006;68:858–61. 17070367 10.1016/j.urology.2006.05.022

